# Self-Efficacy of Saudi Patients with Autoimmune Diseases in Managing Hydroxychloroquine-Induced Ocular Complications: A Cross-Sectional Survey

**DOI:** 10.3390/healthcare10030565

**Published:** 2022-03-17

**Authors:** Amal Aldarwesh, Ali Almustanyir, Duja Alhayan, Mazoon Alharthi, Mohammed Alblowi

**Affiliations:** 1 Department of Optometry and Vision Sciences, College of Applied Medical Sciences, King Saud University, Riyadh 145111, Saudi Arabia; aalmustanyir@ksu.edu.sa (A.A.); 437200037@student.ksu.edu.sa (D.A.); 437202469@student.ksu.edu.sa (M.A.); 2 Department of Optometry, King Khaled Eye Specialist Hospital, Riyadh 11462, Saudi Arabia; malblowi@outlook.com

**Keywords:** self-efficacy, autoimmune disease, systemic lupus erythematosus

## Abstract

Rheumatoid arthritis (RA) and systemic lupus erythematosus (SLE) are common autoimmune diseases (AD) that affect joints and have multi-organ involvement that results in disability, morbidity, and increased mortality. Both conditions are known to cause a wide range of ocular manifestations. Antimalarial drugs, mainly hydroxychloroquine (HCQ), are among the treatment options for AD that is uniquely characterized by retinopathy as a main side effect. This study examines self-efficacy levels in autoimmune disease patients who were or are currently treated with HCQ and related factors such as patient education, communication with the physician, self-education, and ability to cope with the disease.

## 1. Introduction

Rheumatoid arthritis (RA) and systemic lupus erythematosus (SLE) are common autoimmune diseases (AD) that affect joints and have multi-organ involvement that results in disability, morbidity, and increased mortality [1,2]. Coping with chronic debilitating conditions requires that the patient possess good health literacy and self-efficacy skills. Health literacy means that the patient can obtain, process, communicate, and understand basic health information, and act on the information in a way that promotes and maintains a good health state [3]. Patients with low health literacy have been reported to have a higher risk of noncompliance and misunderstanding of medical instructions and, therefore, poor health outcomes [4,5]. Self-efficacy refers to an individual’s confidence about their ability to achieve the desired outcomes when they undertake certain behavior or action [6]. Higher education and health literacy are predictors of better self-efficacy and quality of life in chronic conditions [7,8]. Studies have indicated that participation of SLE patients in educational programs increases their knowledge and improves their coping skills, self-efficacy, and quality of life [9,10,11]. Similarly, higher education in RA patients has been reported to reduce pain and functional disability, and to increase medication adherence [12,13].

As with autoimmune diseases, RA and SLE patients must cope with many aspects of the disease, including organ-specific complications and drug-induced side effects. Both conditions are known to cause ocular involvement in which affected patients may suffer from keratoconjunctivitis sicca, conjunctivitis, keratitis, optic neuritis, and retinopathy [14]. Moreover, some SLE patients may suffer from antiphospholipid syndrome which also leads to high ocular complications such as ocular retinal vascular occlusive disease [15].

Treatment options for autoimmune diseases include non-steroidal anti-inflammatory drugs, immunosuppressants, disease-modifying antirheumatic drugs, and antimalarial drugs, mainly hydroxychloroquine [16]. Despite the effectiveness of these medications in controlling the disease activity, both the disease and treatment can culminate and induce irreversible ocular injury. This scenario is seen with hydroxychloroquine (HCQ), a widely prescribed medication for the long-term management of autoimmunity for its potential therapeutic benefits including immunomodulatory, and antithrombotic effects [16,17]. HCQ-induced ocular toxicity in the form of retinopathy, retinal detachment, and color vision deficiency has been reported [18]. Previous data (unpublished) for Saudi patients with SLE, RA, and other autoimmune diseases who responded to an internet-based survey showed that HCQ is prescribed in higher dosages, which has caused ocular side effects in a small proportion of patients that has necessitated discontinuation. This study examines self-efficacy levels in autoimmune disease patients who were or are currently treated with hydroxychloroquine and related factors such as patient education, communication with the physician, self-education, and ability to cope with the disease.

## 2. Materials and Methods

### 2.1. Study Population

This is a cross-sectional study using an internet-based questionnaire for patients treated with HCQ for the management of ARDs. Patients with SLE, RA, or other autoimmune diseases (*n* = 244), who were previously or currently taking HCQ (Plaquenil^®^) or chloroquine for a period of ≤1 year or >1 year between January and June 2021, were invited to complete an online survey. Participants were recruited in collaboration with the Saudi Society of Rheumatology (SSR, https://www.ssrsa.org/, last accessed on 16 February 2022) and the Charitable Association for Rheumatic Diseases (https://www.rheumatism.sa/, last accessed on 16 February 2022).

### 2.2. Ethical Consideration

The study was approved by the ethical committee of the College of Medicine at King Khalid University Hospital, King Saud University. Informed consent was obtained at the beginning of the online survey. The purpose of the study, age restriction of participants to 18 and above, and their voluntary participation and confidentiality were explained in Arabic in the first page of the questionnaire.

### 2.3. Data Collection

The questionnaire starts with eligibility criteria questions to determine if the participant is over 18 years of old, diagnosed with an autoimmune disease, and using hydroxychloroquine. Then, demographic questions followed (sex, age, level of education). A section related to doctor–patient interaction contained four questions: (1) “Did your doctor provide you orientation about the disease and its possible complications?”, (2) “Did your doctor tell you about the possible side effects of the drug on your eyes?”, (3) “Did your doctor refer you to an ophthalmologist or optometrist for ocular examination at the start of the treatment with hydroxychloroquine?, and (4) “Did you know it is necessary to be accompanied by an ophthalmologist or optometrist at least once per year?” The scale for self-efficacy contained six sentences that measured the confidence of patients in understanding available information about the disease or treatment, communicating with their physician, judging the condition of the eye, and coping with the disease. Patients were asked to indicate their level of confidence about each sentence (1 = not at all confident, 2 = not confident, 3 = fairly confident, 4 = confident, and 5 =completely confident). Higher scores indicated higher levels of self-efficacy. Computing Cronbach’s alpha and average inter-item correlation between the six questionnaire items determined the internal consistency of the questions and showed that the items of the scale correlated to the extent to which the items measure the same construct. A Cronbach’s alpha of 0.70 or greater and item correlation between 0.15 and 0.5 are generally accepted to reflect good internal consistency and correlation.

### 2.4. Statistical Analysis

Descriptive statistics were calculated. Continuous variables were presented as mean values ± standard deviation (SD), whereas categorical variables were presented as absolute and relative frequencies.

The association between the level of confidence and the answers to questions in the questionnaire was found using Chi-square or Fisher’s test as appropriate. The summated subscale scores for completely confident/confident were combined, and the similarly not confident/not at all confident were treated as one category. The result was considered significant at *p* < 0.05.

## 3. Results

### 3.1. Internal Consistency Reliability and Inter-Item Correlation

Internal consistencies of the questions were determined using Cronbach’s alpha. Cronbach’s alpha for the total scale was 0.80, indicating a good estimate of internal consistency reliability. The mean inter-item correlation was 0.40, indicating a good correlation of all items in the scale. Details of item analysis are shown in Table 1.

### 3.2. Characteristics of Participants

Of the 414 respondents, 244 patients completed the survey and were eligible to be included. Most were female (94.3%), and the majority of the sample was diagnosed with SLE (59.4%). The rest were diagnosed with rheumatoid arthritis (30%) and other autoimmune diseases (10.7%), such as Sjögren’s syndrome, mixed connective tissue disease, antiphospholipid syndrome, and immune thrombocytopenia (ITP). Most of the patients had received a bachelor’s degree (57.4%). Their ages ranged from 18 to 75 years (mean = 35.6, SD = 10), with an average duration of the disease of 5.3 years (SD = 5.34). The characteristics of the study participants are shown in Table 2.

### 3.3. Self-Efficacy Analysis

The level of confidence for each item on the five-point Likert scale is presented in Table 3. Examining the level of education with the confidence level about retrieving information from Arabic resources, those who were educated and held a bachelor’s degree or higher did not show higher efficacy compared to lower education levels. However, an overall score on five-point Likert scale was relatively low for each disease category in which SLE, RA, and other AD patients reported 3.4, 3.5, and 2.8, respectively (Table 3). Data were stratified according to the age and level of education in Table 4. Patients who were provided with information about the disease and its possible complications by their treated physician were more confident about having deep knowledge about their condition (Chi-square = 25.63, *p* < 0.0001), and had high confidence about communicating their concerns to the doctor (Chi-square = 23.6, *p* < 0.0001). Similarly, patients who were educated about the ocular side effect of HCQ at the beginning of the treatment felt confident about having a deep understanding of their condition (Chi-square = 29.4, *p* < 0.0001). A slightly lower confidence level was reported for self-education and reading information about the disease or medication from Arabic resources. The level of confidence for those who were referred to an ophthalmologist or optometrist for ocular examination at the start of the treatment was similar to patients with no referral. Those who knew it was necessary to see an ophthalmologist or optometrist during the therapy were also more confident about the health benefits to their sight (Fisher’s exact test statistic value: 0.0327. *p* < 0.05). Approximately 70% of patients were confident that they were able to judge when changes in the eye required visiting the clinic and coping with the disease.

## 4. Discussion

This research explores the level of self-efficacy in Saudi patients affected by SLE, RA, and other autoimmune diseases. Patients were asked to rate their confidence level concerning communication with physicians, self-education, and judgment of their ocular condition, and coping with the chronic illness. Self-efficacy has been proven to impact the management of many chronic conditions, such as cardiovascular and respiratory diseases, diabetes, cancer, and autoimmune disease [16,17].

Effective patient–physician interaction and education level have been known to improve health care outcomes through enhancing patients’ understanding of the disease and adherence to medications. In a study of patients’ satisfaction about health education, those who received direct medical information from their physicians were more satisfied with the care they received and felt empowered [18,19,20].

This is in accordance with current data showing that patients with autoimmune disease who received disease- and drug-related information had more confidence and empowerment. Currently, patients of all ages can access health-related information through the internet and social media, and they may rely on that for self-education. Patients were asked about their confidence about retrieving knowledge about their disease and treatment from Arabic resources, and they revealed a slightly lower confidence level. This score could be attributed to the fact that the most trustworthy and available scientific literature comes from English resources.

On the other hand, studies have reported lower readability and quality of information on Arabic websites [21]. This may impose a language barrier for disadvantaged individuals who are older or those with lower educational levels. Therefore, those individuals are considered to have low health literacy. Given the importance of education, Jiang et al. (2015) [12] found that higher educated, newly diagnosed RA patients have less pain and functional disabilities, and are more likely to go into remission than lower educated people.

This may indicate that education is a factor in developing patients’ self-management and self-efficacy skills. The majority of the participants held a university/college degree. When the efficacy level was compared to lower educational levels, they did not significantly differ in confidence. However, the number of patients with lower educational status (high school, elementary, and primary) were low in number. Therefore, it was difficult to conclude the relationship between self-efficacy and education level. Patients in this study did not entertain serious doubts about coping with the chronic disease and its symptoms. According to Bandura (1977) [6], efficacy expectation is the contentment that one can successfully carry out a behavior required to produce the outcomes. Although not measured here, religious beliefs could influence the individuals’ acceptance of the disease and its effects, perceiving it as part of their fate predestined by God [22].

## 5. Conclusions

Current findings suggest that Saudi patients with autoimmune diseases in different age groups and educational levels have a good level of self-efficacy. Identifying patients in need of self-management and providing support from the physician can improve health outcomes and reduce morbidities in chronically individuals. Further study with a larger sample size is necessary to assess the efficacy level for patients with lower educational levels.

## Figures and Tables

**Table 1:** Item analysis of the self-efficacy questions (*n* = 244).

Inter-Item Correlation						Average Inter-Item Correlation	Cronbach’s Alpha
Q1	Q2	Q3	Q4	Q5	Q6		
0.383	0.402	0.433	0.444	0.395	0.371	0.404	0.80

**Table 2:** Demographics and patients’ characteristics (*n* = 244).

	Condition	SLE	RA	Other ADs
**Patient Characteristics, No. (%)**				
	*n*	145	73	26
**Gender**	Male	6 (4.1)	6 (8.2)	1 (3.8)
	Female	139 (95.9)	67 (91.8)	25 (96.2)
**Education**	Primary School	3 (2.1)	1 (1.4)	1 (3.8)
	Secondary School	5 (3.4)	1 (1.4)	2 (7.7)
	High School	21 (14.5)	19 (26)	4 (15.4)
	Diploma	14 (9.7)	5 (6.8)	3 (11.5)
	Bachelor	81 (55.9)	44 (60.3)	15 (57.7)
	Higher Education	20 (13.7)	3 (4.1)	2 (7.7)
**Age (years), mean (SD)**		33.4 (9.3)	39 (10.4)	37.5 (10.3)
**Duration of the disease (years), mean (range)**		7.2 (0.08–35)	6.5 (0.16–28)	5.9 (1–26)

Abbreviations: SLE, systemic lupus erythematosus; RA, rheumatoid arthritis; ADs, autoimmune diseases.

**Table 3:** Self-efficacy of patients with autoimmune diseases. Score obtained from 5-point Likert scale (%), *n* = 244.

Sentences	SLE	RA	ADs	Overall Score
	*n* = 145	*n* = 73	*n* = 26	
	Mean Score ± SD (%)			
Q1. How confident are you that you can get information about your disease or medication from scientific/medical resources that are available in Arabic?	3.4 ± 1.2	3.5 ± 0.9	2.8 ± 1.2	3.4 ± 1.1
	(67.2)	(69.3)	(57.7)	(68.10)%
Q2. How confident are you in your deep knowledge of your disease and its effect on the eye?	3.2 ± 1.2	3.5 ± 3.2	2.7 ± 1.2	3.2 ± 1.2
	(64.7)	(62.5)	(54.5)	(63.1)
Q3. How confident are you that you can ask your doctor things about your illness that concerns you?	3.8 ± 1.2	3.6 ± 1.1	3.5 ± 1.3	3.7 ± 1.2
	(75.4)	(71.2)	(72.3)	(73.8)
Q4. How confident are you that you can judge when changes in your eyes mean should visit an ophthalmologist or optometrist?	3.7 ± 1.2	3.8 ± 0.9	3.3 ± 1.2	3.7 ± 1.1
	(73.9)	(76.7)	(67.7)	−74
Q5. How confident are you that it will help your vision to come to appointments with an ophthalmologist or optometrist?	4.0 ± 1.1	3.9 ± 0.8	3.6 ± 1.3	4.0 ± 1.02
	(80.8)	(78.1)	(72.3)	−79
Q6. How confident are you in your ability to cope with your chronic disease and its symptoms?	3.8 ± 1.2	3.9 ± 0.9	3.5 ± 1.0	3.8 ± 1.1
	(76.8)	(78.1)	(71.5)	(76.7)

**Table 4:** Confidence level according to age and level of education.

	Age	Q1	Q2	Q3	Q4	Q5	Q6
Higher Education (*n* = 25)							
Min	23	1	1	1	2	1	1
Max	53	5	5	5	5	5	5
Average	35.9	3.2	3.1	3.8	3.5	3.8	3.7
SD	8	1.05	0.95	1.19	1.00	1.26	1.21
Bachelor (*n =* 140)							
Min	20	1	1	1	1	2	1
Max	62	5	5	5	5	5	5
Average	35.3	3.5	3.1	3.7	3.7	4.0	3.9
SD	9.5	1.0	1.1	1.2	1.2	0.9	1.1
Diploma (*n* = 22)							
Min	19	1	1	1	1	2	1
Max	55	5	5	5	5	5	5
Average	34	3.3	3.0	3.9	3.8	4.0	3.6
SD	10.2	1.20	1.40	1.02	1.05	1.07	1.00
High School (*n* = 44)							
Min	18	1	1	1	1	1	1
Max	58	5	5	5	5	5	5
Average	34.6	3.3	3.4	3.7	3.9	4.1	3.8
SD	9.7	1.2	1.3	1.3	1.0	1.0	1.3
Secondary (*n* = 8)							
Min	18	1	3	3	2	1	2
Max	60	5	5	5	5	5	5
Average	41.5	3.0	3.6	3.8	4.0	3.3	4.0
SD	13.7	1.1	0.9	0.9	1.1	1.8	1.2
Primary School (*n* = 5)							
Min	27	1	1	1	1	3	3
Max	75	4	4	5	4	4	5
Average	50.3	2.5	2.3	3.5	2.8	3.5	4.3
SD	19.7	1.3	1.5	1.7	1.3	0.6	1.0

## Data Availability

Not applicable.
